# Adverse perinatal outcomes among children in Switzerland: the impact of national origin and socio-economic group

**DOI:** 10.1007/s00038-020-01492-2

**Published:** 2020-10-04

**Authors:** Philippe Wanner

**Affiliations:** Institute of Demography and Socioeconomics, NCCR – on the Move, University of Geneva, Geneva, Switzerland

**Keywords:** Infant mortality, Low birth weight, Preterm birth, Migrants’ children, Health inequalities, Switzerland

## Abstract

**Objectives:**

We examined the effect of the mother’s origin and socio-economic characteristics on adverse perinatal outcomes in Switzerland.

**Methods:**

Births occurring from 2011 to 2017 were identified in the Swiss population register and merged with the Swiss civil register and the Register of the first pillar to obtain information on the migration origin and socio-economic level. Four indicators of adverse perinatal outcomes were defined.

**Results:**

Logistic regressions show that both the migration origin and the socio-economic level are measured by the parents’ income, influence risk. Compared to the children of mothers born in Switzerland, those of mothers from EU/EFTA countries have a lower risk of infant mortality, low birth weight and extreme prematurity. The highest risk is observed for children born to mothers from the rest of the world. High levels of risk consistently characterize children with low-income parents (first decile).

**Conclusions:**

Our results justify further investigations at the level of health services to better identify the factors causing differences in the prevalence of adverse outcomes and to take them into account in adapted health policies.

**Electronic supplementary material:**

The online version of this article (10.1007/s00038-020-01492-2) contains supplementary material, which is available to authorized users.

## Introduction

Switzerland has one of the highest levels of life expectancy at birth in the world. In 2017, according to the WHO Global Health Observatory (2020), it ranked second behind Hong Kong (81.7 years) for men and seventh for women (85.7 years). Paradoxically, in the same year, Switzerland was ranked 32nd in the world in terms of infant mortality, with an infant death rate per 1000 births higher than the EU average (3.7‰ compared to 3.3‰) and higher than that of all its neighbouring countries (Italy 2.7‰, Austria 2.9‰, Germany 3.2‰ and France 3.4‰—WHO 2020). Over the past decades, the level of this indicator has also fallen more slowly than that in neighbouring countries (between 2000 and 2017, a decrease of 21%, compared to 23–43% in the four neighbouring countries). The percentage of low-weight (< 2500 g) births has been estimated as 6.5% (Blencowe et al. 2019), a value that is average for industrialized countries. However, this value has increased by half a point since 2000. The percentage of preterm live births (22–36 weeks) was 7.2% in 2015, which is close to that of neighbouring countries (Euro Peristat 2015). Paradoxically, while the Swiss have an extremely low overall mortality rate, child health indicators are at average levels.

This paradox has been analysed by Wanner and Bollini (2017), who attribute it to births occurring in high-risk migrant communities (especially among those of Turkish and Spanish origin). According to Bollini et al. (2009), the Swiss health system has had difficulty accommodating the increasing diversity of migrant populations, particularly in ensuring optimal communication between mothers who do not speak a Swiss language and health care providers. Such excess mortality of children of migrant women is not specific to Switzerland and has been observed in other countries of immigration, such as Italy (Simeoni et al. 2019, for migrants of central and South African origin) and the United States (Singh and Yu 1996). It is sometimes linked to the care provided by the health system to migrant communities but can also be explained by social and economic factors specific to migrant populations (precarious living conditions, heavy work during pregnancy, etc.) that lead to increased stress during pregnancy. These same socio-economic factors have been shown to lead to health inequalities well after birth, during childhood or adolescence (Jaeger et al. 2012).

Trends in child mortality in Switzerland have recently been studied (Junker and Berrut 2011) and analysed from the perspective of the child’s nationality (Wanner and Bollini 2017). However, the impact of the parents’ socio-professional situation on the outcome of pregnancy and birth is not known. This is due to the lack of data. However, it has been shown elsewhere that socio-economic or professional status plays a role in infant mortality (Rosenquist et al. 2020 in the United States; Fell et al. 2000 in Canada) and in other perinatal outcomes, such as preterm birth (Chiavarini et al. 2012; Puthussery et al. 2019).

The socio-economic structure of the population varies according to origin. In Switzerland, the population of foreign origin is present in different sectors of activity at both ends of the social scale. An over-representation of nationals from states belonging to the European Union and the European Free Trade Association (EU/EFTA) and of nationals from the OECD can be observed among managers and in positions of responsibility, particularly in the service sector. However, a large Portuguese and non-EU population participates in low-paid service or construction activities. For this reason, unfavourable child health indicators for children of foreign origin may reflect the effects of socio-economic status. Using original data, this article extends the analyses already carried out on the Swiss paradox by integrating data on the socio-economic dimension into the analyses. The aim is to discuss the respective impacts of the migration trajectory and the economic situation on the risk of an unfavourable perinatal outcome. In addition to infant mortality, three other pregnancy outcomes are studied.

## Methods

The data are extracted from a matched longitudinal database prepared as part of a national migration research programme (NCCR on the Move—Steiner and Wanner 2015). These data make it possible to link several administrative registers and surveys. We extracted live births and infant deaths recorded in the Swiss population statistics (STATPOP) for the period 2011–2017. These statistics are based on the harmonized register of persons, which came into force at the end of 2010. Live births include births of children who, after complete expulsion (head, trunk, limbs) from the mother’s body, are breathing or in whom at least a heartbeat is observed. Births were matched with the civil register to retrieve information on the status of the birth (birth weight, gestation duration) and on the parents (age, marital status). A deterministic matching was carried out using the anonymous social security number (Old-age and Survivors’ Insurance Number - NAVS13) that appears in all official administrative registers in Switzerland. All persons legally resident in Switzerland are assigned a single NAVS13. For this reason, the matching rate between administrative registers is considered to be 100%.

The register of live births also provides the NAVS13 identifier of both parents. Based on this identifier, we searched for various data on the father and mother in the register of the Swiss Central Compensation Office, which provides the annual professional income of each person with a declared professional activity in Switzerland.

These various registers, which we matched, made it possible to create a dataset that includes all 590,697 live births in Switzerland between 2011 and 2017.

Four adverse perinatal outcomes were analysed: infant mortality (IM), defined here as the death of a child occurring the same year as the birth year; very low birth weight (VLBW, < 1500 g); preterm birth (PTB, < 37 weeks of gestation); and extreme preterm birth (EPTB, < 28 weeks of gestation).

Logistic regressions were run to measure the association of different risk factors with the different outcomes. Logistic regressions were performed to explain the probability (*p*) of one perinatal outcome according to the dimensions under study and several control variables (Cox and Snell 1989). The formula is as follows:

$$ \log {\text{it}}\left( p \right) = \ln \left( {p/\left( {1 - p} \right)} \right) = \beta \_0 + \beta \_1 \, x\_\left( {i,1} \right) + \beta \_2 \, x\_\left( {i,2} \right) + \cdots $$

where *β*_0 is a constant and *β*_(1,…*n*) are the coefficients of the explanatory variables *x*_(1,… *n*). The exponential value of *β*_(1,…*n*) is the odds ratio.

For all models, the levels of significance (*p < 0.05; **p < 0.01; ***p < 0.001) are presented to facilitate the interpretation of the results.

The models include the following mother and child variables:

- Multiparity: single birth (reference category), multiple birth;
- Mother’s age in three categories: < 20, 20–34 (reference category), and 35 +;
- Mother’s marital status in two categories: married (reference category, including persons living in legal partnership) and unmarried.

The variables associated with migratory origin are as follows:

- Mother’s country of birth: Switzerland (reference category), EU/EFTA countries, other OECD countries (except Turkey) and other non-OECD countries (“rest of the world”, including Turkey);
- Residence status: Switzerland (reference category), settlement permit, annual permit, asylum-related permit and other permit (mainly short-term permits).

Finally, the highest income obtained by either parent in the 3 years before birth, was distributed in deciles. The highest income is generally used as an indicator of household financial capability, but also as an indicator of the level of integration in the labour market for foreign-born populations (see Wanner 2020). The first decile includes relatively young, unmarried mothers from the rest of the world. It includes most (nearly 90%) of the asylum seekers.

The categorization of countries of origin takes into account differences in migration policies (residence in Switzerland is generally more precarious for people from outside the EU/EFTA) and the economic situation of the respective migrant communities (see, for instance, D’Amato et al. 2019).

## Results

### Sample description

Table 1 presents the sample and incidence rates (per 1000) of the different outcomes. A total of 3.4‰ of live-born children died in the year of birth, nearly 10‰ had VLBW, 8% were born before 37 weeks of gestation and 4‰ were born before 28 weeks of gestation. As expected, the frequency of adverse perinatal outcomes is higher among unmarried mothers, which is probably partly related to an age effect. Pregnancies that occur in young mothers (under 20 years of age) and older mothers (35 years of age and older) are characterized by the most unfavourable perinatal outcomes. Moreover, the frequency of adverse outcomes depends closely on the type of birth (single or multiple).

**Table 1:** Description of the sample of mothers who have given birth between 2011 and 2017 (Switzerland 2011–2017). *Sources*: Swiss Population Statistics/Swiss civil register/Central Compensation Office register

	IM		VLBW		PTB		EPTB		*N*
	*n*	*p*. 1000	*n*	*p*. 1000	*n*	*p*. 100	*n*	*p*. 1000	
Total	1997	3.4	5797	9.8	46,988	8.0	2383	4.0	590,697
*Marital status*									
Married	1437	3.1	4085	8.9	35,785	7.8	1651	3.6	460,123
Not married	560	4.3	1712	13.1	11,203	8.6	732	5.6	130,574
*Age of mother*									
< 20	21	3.8	73	13.2	438	8.0	28	5.1	5517
20–34	1366	3.2	3752	8.9	31,201	7.4	1613	3.8	421,429
35 +	610	3.7	1972	12.0	15,349	9.4	742	4.6	163,751
*Multiple pregnancy*									
No	1664	2.9	3797	6.7	34,041	6.0	1778	3.1	569,241
Yes	333	15.5	2000	93.2	12,947	60.6	605	28.3	21,456
*Mother’s country of birth*									
Switzerland	1038	3.2	3058	9.4	25,842	8.0	1155	3.6	323,665
UE/EFTA	333	3.0	974	8.8	8822	8.0	382	3.5	110,642
Other OECD	24	2.5	95	9.8	761	8.0	31	3.2	9653
Other non-OECD	602	4.1	1670	11.4	11,563	7.9	815	5.6	146,737
*Permit of residence*									
Swiss	1202	3.2	3561	9.6	29,923	8.1	1403	3.8	370,500
Annual permit	406	3.6	1058	9.4	8218	7.4	487	4.4	112,107
Permanent permit	347	3.7	1022	10.8	7861	8.3	433	4.6	94,935
Asylum seeker	20	3.2	76	12.1	410	6.5	27	4.3	6275
Other permit	22	3.2	80	11.6	576	8.4	33	4.8	6880
*Income (range in Swiss francs)*									
First decile (< 46,084)	349	5.0	921	13.2	5731	8.3	426	6.2	69,579
Second decile (46,084–59,071)	209	3.6	588	10.1	4390	7.6	260	4.5	57,985
Third decile (59,072–67,066))	210	3.6	574	9.9	4359	7.5	245	4.2	58,105
Fourth decile (67,067–74,060)	209	3.6	583	10.0	4479	7.7	249	4.3	58,125
Fifth decile (74,061–81,480)	180	3.1	514	8.9	4449	7.7	177	3.1	57,929
Sixth decile (81,481–90,370)	145	2.5	493	8.5	4411	7.6	194	3.4	57,825
Seventh decile (90,371–101,646)	157	2.7	492	8.5	4502	7.8	174	3.0	57,729
Eighth decile (101,647–117,939)	170	2.9	509	8.8	4557	7.9	206	3.6	57,643
Ninth decile (117,940–149,749)	194	3.4	556	9.6	4772	8.3	229	4.0	57,838
Tenth decile (149,750 +)	174	3.0	567	9.8	5338	9.3	223	3.9	57,939

*IM* infant mortality, *VLBW* very low birth weight (< 1500 g); preterm birth (< 37 weeks of gestation), *EPTB* extreme preterm birth (< 28 weeks of gestation)

Descriptive analyses also show an increased risk for children of mothers from countries in the rest of the world, mainly with respect to infant mortality, low birth weight and EPTB. The differences observed among children of mothers from EU/EFTA countries, other OECD countries and Switzerland are small and differ by indicator. With regard to residence permits, the risk of infant mortality and EPTB is higher for children of mothers with annual or settlement permits than for Swiss mothers. A high incidence of VLBW and EPTB also characterizes women with asylum-related or other permits.

Finally, children whose parents have an income in the first decile perform very poorly on all indicators. On the other hand, children of parents whose income is above the median do well except with respect to prematurity.

### Multivariate analysis

The logistic regressions conducted on the different outcomes yield results consistent with expectations for the variables related to the characteristics of the birth and the mother. Marital status has an impact on the four indicators analysed, since the children of unmarried mothers are at greater risk of low birth weight, prematurity or infant death. This result can be explained in part by the socio-professional and family context in which pregnancy occurs for some unmarried mothers. This may also be due because marital status partly captures the effect of age, as unmarried women are over-represented at both ends of the reproductive timeline.

A maternal age of 35 years or older significantly increases the risk of a negative perinatal outcome, whereas an age below 20 years—which is rare in Switzerland—leads only to a significant increase in PTB. Finally, as expected, multiple births lead to a significantly increased risk of infant mortality and an even greater increase in the risk of low birth weight and prematurity.

Once these biological and marital status factors are controlled, the mother’s place of birth always has an impact on the risk of infant mortality. Compared to the children of mothers born in Switzerland, those of mothers from EU/EFTA countries have a lower risk of infant mortality (OR 0.83 [0.71–0.97]), very low birth weight (OR 0.84 [0.77–0.91]) and extreme prematurity (OR 0.87 [0.76–1.00]). A lower risk of negative outcomes also appears for women from non-European OECD countries, but it is not significant. However, for three of the four indicators (IM, VLBW and PTB), children born to mothers from the rest of the world are at greater risk than those born to Swiss women at birth. This risk is especially significant for prematurity (1.49 [1.33–1.67]).

With regard to the impact of the residence permit, there is no increase in risk among foreign populations with short-term permits. A surprising result is observed with respect to the low prevalence of infant mortality (0.57 [0.36–0.91]) and prematurity (PTB 0.74 [0.66–0.83]; EPTB 0.54 [0.36–0.81]) among women with an asylum seekers or provisional admission permit. The risk of PTB is also low for women with an annual permit (0.93 [0.90–0.96]).

High levels of risk consistently characterize children with low-income parents (first decile). It is especially for the most severe outcomes (EPTB, 1.74 [1.45–2.09]; IM, 1.55 [1.28–1.87]) that the differences in odds ratios are the greatest.

For children of parents whose income is between the second and fourth deciles, the risks are also significantly higher than for those with parents in the fifth decile with respect to VLBW and EPTB. For the latter indicator, the risk seems to change from one decile to another in a U-shaped pattern, since after reaching a nadir in the median deciles, it increases slightly for the upper deciles.

**Fig. 1:**
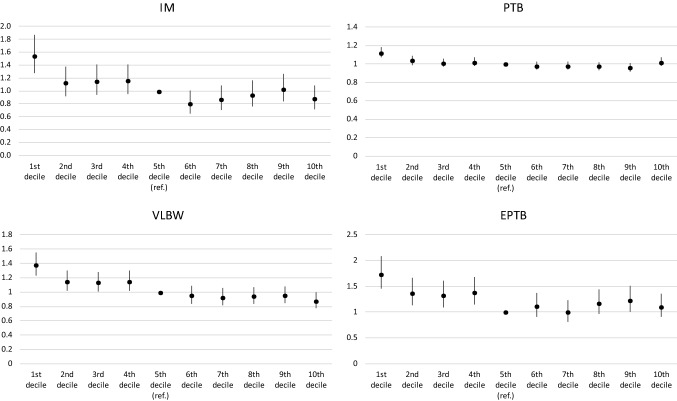
Odds ratios and confidence intervals by decile of parental income resulting from the logistic regressions for the different outcomes (Switzerland 2011–2017). IM = Infant mortality; VLBW = Very low birth weight (< 1500 g); preterm birth (< 37 weeks of gestation); EPTB = extreme preterm birth (< 28 weeks of gestation). *Sources*: Swiss Population Statistics/Swiss civil register/Central Compensation Office register

Figure 1 shows the evolution of the odds ratios presented in Table 1 for the four perinatal outcomes by income decile. Systematically, except for the indicators of prematurity, a decreasing risk gradient is observed as the parental income decile increases. For PTB, the odds ratio holds steady from the second decile onwards.

### Impact of full coverage of prenatal examination costs

Since 1 March 2014, the Swiss health system has required no deductible or contribution towards the costs of childbirth and basic prenatal examinations. Previously, women were required to contribute to these medical costs. This situation may have led to less consultation seeking among women from the most precarious social classes, i.e. those in the first deciles of income and those from countries in the rest of the world. The new 2014 provision was intended to rectify this pattern. However, it is not known to what extent the women concerned have been informed of this new provision. In addition, a journalistic study in 2017 indicated that some insurance companies “forgot” this clause and continued to charge a participation fee.

**Fig. 2:**
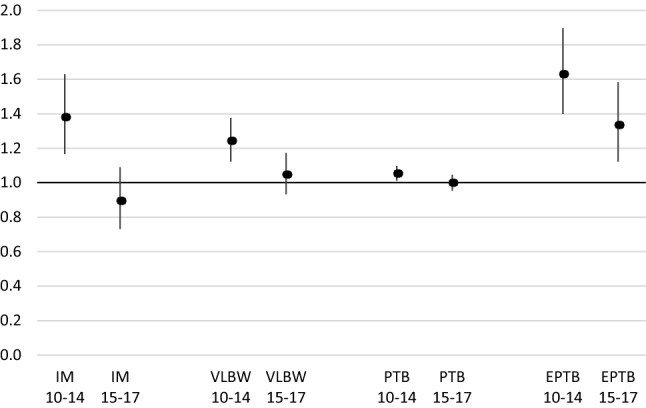
Odds ratios and confidence intervals (95%) for mothers in the rest of the world for the four selected indicators in 2010–2014 and 2015–2017 (Switzerland 2011–2017). IM = Infant mortality; VLBW = Very low birth weight (< 1500 g); preterm birth (< 37 weeks of gestation); EPTB = extreme preterm birth (< 28 weeks of gestation). *Sources*: Swiss Population Statistics/Swiss civil register/Central Compensation Office register

To test the impact on children’s health of the change in the level of reimbursement of medical expenses, logistic models were run with a distinction made between births occurring between 2011 and 2014 and those occurring between 2015 and 2017. In general, the results presented in Table 2 are confirmed for both periods. However, as Fig. 2 shows, complete reimbursement has resulted in a decrease in outcome differentials between the children of women born in countries in the rest of the world and those born to Swiss women.

**Table 2:** Results of the logistic regressions on the four pregnancy outcomes among live births (Switzerland 2011–2017). *Sources*: Swiss Population Statistics/Swiss civil register/Central Compensation Office register

	IM (*n* = 1997)				VLBW (*n* = 5797)				PTB (*n* = 46,988)				EPTB (*n* = 2383)			
	O.R.	C.I.		Sign	O.R.	Confidence limits		Sign	O.R.	Confidence limits		Sign	O.R.	Confidence limits		Sign
*Marital status*																
Married (ref.)	1.00				1.00				1.00				1.00			
Not married	1.41	{1.27	− 1.56}	***	1.60	{1.50	− 1.70}	***	1.20	{1.17	− 1.23}	***	1.71	{1.56	− 1.87}	***
*Age of mother*																
< 20	0.84	{0.54	− 1.31}		1.21	{0.95	− 1.54}		1.11	{1.00	− 1.23}	*	0.95	{0.65	− 1.39}	
20–34 (ref.)	1.00				1.00				1.00				1.00			
35+	1.16	{1.05	− 1.28}	**	1.24	{1.17	− 1.31}	***	1.16	{1.14	− 1.19}	***	1.13	{1.03	− 1.24}	**
*Multiple pregnancy*																
No (ref.)	1.00				1.00				1.00				1.00			
Yes	5.51	{4.89	− 6.20}	***	15.78	{14.91	− 16.71}	***	24.10	{23.40	− 24.83}	***	9.58	{8.72	− 10.53}	***
*Mother’s country of birth*																
Switzerland (ref.)	1.00				1.00				1.00				1.00			
UE/EFTA	0.83	{0.71	− 0.97}	*	0.84	{0.77	− 0.91}	***	0.99	{0.96	− 1.02}		0.87	{0.76	− 1.00}	*
Other OECD	0.71	{0.47	− 1.08}		0.96	{0.78	− 1.19}		0.93	{0.85	− 1.01}		0.85	{0.59	− 1.22}	
Other non-OECD	1.15	{1.01	− 1.30}	*	1.15	{1.07	− 1.24}	***	1.03	{1.00	− 1.06}		1.49	{1.33	− 1.67}	***
*Permit of residence*																
Swiss (ref.)	1.00				1.00				1.00				1.00			
Annual permit	1.11	{0.97	− 1.28}		1.04	{0.96	− 1.14}		0.93	{0.90	− 0.96}	***	1.06	{0.93	− 1.20}	
Permanent permit	1.09	{0.95	− 1.25}		1.08	{0.99	− 1.17}		0.99	{0.95	− 1.02}		1.06	{0.94	− 1.20}	
Asylum seeker	0.57	{0.36	− 0.91}	*	0.86	{0.67	− 1.09}		0.74	{0.66	− 0.83}	***	0.54	{0.36	− 0.81}	**
Other permit	0.85	{0.55	− 1.31}		1.12	{0.88	− 1.41}		1.02	{0.93	− 1.13}		1.03	{0.72	− 1.47}	
*Income (range in Swiss francs)*																
First decile (< 46,084)	1.55	{1.28	− 1.87}	***	1.38	{1.23	− 1.55}	***	1.12	{1.07	− 1.18}	***	1.74	{1.45	− 2.09}	***
Second decile (46,084–59,071)	1.13	{0.92	− 1.38}		1.15	{1.02	− 1.30}	*	1.04	{0.99	− 1.09}		1.37	{1.13	− 1.66}	**
Third decile (59,072–67,066))	1.15	{0.94	− 1.41}		1.14	{1.01	− 1.28}	*	1.01	{0.97	− 1.06}		1.33	{1.09	− 1.61}	**
Fourth decile (67,067–74,060)	1.16	{0.95	− 1.41}		1.15	{1.02	− 1.30}	*	1.02	{0.98	− 1.07}		1.39	{1.14	− 1.68}	***
Fifth decile (74,061–81,480)	1.00				1.00				1.00				1.00			
Sixth decile (81,481–90,370)	0.81	{0.65	− 1.01}		0.96	{0.84	− 1.09}		0.98	{0.94	− 1.03}		1.12	{0.91	− 1.37}	
Seventh decile (90,371–101,646)	0.87	{0.70	− 1.08}		0.93	{0.82	− 1.06}		0.98	{0.94	− 1.03}		1.00	{0.81	− 1.23}	
Eighth decile (101,647–117,939)	0.94	{0.76	− 1.16}		0.95	{0.84	− 1.07}		0.98	{0.93	− 1.02}		1.17	{0.96	− 1.44}	
Ninth decile (117,940–149,749)	1.03	{0.84	− 1.27}		0.96	{0.85	− 1.08}		0.96	{0.92	− 1.01}		1.23	{1.01	− 1.51}	*
Tenth decile (149,750 +)	0.88	{0.71	− 1.09}		0.88	{0.78	− 1.00}	*	1.02	{0.98	− 1.07}		1.11	{0.91	− 1.36}	
Somers’ D	0.21				0.42				0.31				0.35			
Gamma	0.43				0.52				0.33				0.50			
Tau-a	0.00				0.01				0.05				0.00			
c	0.60				0.71				0.65				0.68			
Likelihood Ratio	698.44	< .0001			6715.3	< .0001			42,025.8	< .0001			1790.9	< .0001		
Score	1147.41	< .0001			16,427.9	< .0001			84,014.9	< .0001			3594.2	< .0001		
Wald	943.62	< .0001			9501.4	< .0001			44,726.1	< .0001			2521.5	< .0001		
Observations	590,697				590,697				590,697				590,697			

*IM* infant mortality, *VLBW* very low birth weight (< 1500 g); preterm birth (< 37 weeks of gestation), *EPTB* extreme preterm birth (< 28 weeks of gestation)

*p < 0.05; **p < 0.01; ***p < 0.001

On the other hand, the new provision did not result in a significant change in the outcomes of women in the lowest decile (results not shown). It is likely that for women in a particularly precarious financial situation, other factors (related to lifestyle or arduous work) influence the perinatal outcome.

## Discussion

In Switzerland in 2017, infant mortality is still higher in Switzerland than in neighbouring countries. This article discusses whether socio-economic factors or factors linked to migrant populations are likely to explain this situation. The analyses carried out here show that after controlling for biological factors, the risks of an unfavourable outcome are higher for women from the rest of the world and for those with low incomes.

The integration into the models of these two dimensions—national origin and financial resources, indicators of socio-professional position—confirms the results of Wanner and Bollini (2017) on the high risk for certain groups of migrants. This increased risk is verified by multivariate models after taking income into account. In addition, the results show for the first time in the case of Switzerland an increased risk for women from a couple with low financial resources, irrespective of origin.

In Switzerland, migration has a dual nature, with the arrival of a highly qualified population from EU/EFTA countries, with the exception of Portugal, and from OECD countries (D’Amato et al. 2019). Migrant populations from the rest of the world have a more variable profile in terms of educational attainment, since highly qualified persons and students arriving for tertiary or vocational training purposes rub shoulders with low-skilled persons who have arrived for asylum or for family reunification. In this context, the low levels of risk for children of women from EU/EFTA and OECD countries can be explained by optimal levels of education and living conditions.

Our results show an increased risk for other migrant communities, probably linked to the difficulty for health policies in the host country of taking these populations into account (Bollini et al. 2009; Naimy et al. 2013). In particular, communication difficulties between health workers and migrant populations of diverse backgrounds and cultures may play a role in promoting negative outcomes. Maternal living conditions, particularly working conditions, longer commuting distances, or inappropriate health behaviours, are also likely to influence prematurity and the incidence of low birth weight. However, the effects of these factors are hypothetical and should be investigated in more detail in qualitative studies.

The fact that the residence permit does not act as a risk factor after origin is taken into account is explained by the fact that access to health care is guaranteed for all immigrant populations, regardless of their residence status in Switzerland. It is also possible that the fact that recently arrived migrants (permit B) are selected (by the so-called healthy migrant-effect, which has been proven to play a role on perinatal outcomes—Wingate and Alexander 2006) reduces their risk compared to the reference population. The reduced risk for children of women with an asylum permit is, however, a counter-intuitive result. These women benefit from basic medical care but encounter obstacles at the time of pregnancy. According to Cignacco et al. (2018), they are indeed subject to structural restrictions in the organization of care, which is often fragmented and decentralized, and to disruptions in the provision of care. One would therefore expect less favourable perinatal outcomes. There is evidence, however, that these women have difficulty integrating professionally (Spadarotto et al. 2014; Hainmueller et al. 2016), which could keep them from experiencing the excessive professional burdens carried by women of the same origin holding another permit.

With regard to the migrant women most at risk, our results show that the introduction in 2014 of the full reimbursement of prenatal care by insurance companies was accompanied by a reduction in relative risk for the children of women from the rest of the world compared to those born in Switzerland, attesting to the value of reducing barriers to prenatal consultations to ensure favourable perinatal outcomes. However, a decrease in risk was not observed for the children of women with very low incomes, suggesting that alternative birth support mechanisms are needed for this group.

Our study has some limitations. On the one hand, only births occurring among the resident population are considered. Due to a lack of data, we cannot consider the population living without a residence permit in Switzerland. However, if this population is relatively small (50,000–100,000 people would be in this situation), it could present specific risks. On the other hand, the measurement of the impact of socio-economic and migratory factors on the risk related to pregnancy and childbirth is limited by the availability of data. For example, there is no register in Switzerland indicating the level of education or socio-economic status, which forced us to use a proxy indicator, professional income, which may be imperfect. The father’s origin was also missing for part of the births and therefore not taken into account. We are also aware of the fact that other population-related or medical (i.e. the use of assisted reproductive technology) factors can impact some of the outcomes, such as PTB. Finally, it is important to remember that we had to restrict infant mortality to deaths occurring in the year of birth, which deviates from the generally accepted (age-based) definition. In a country where the majority of child deaths occur in the early neonatal period, however, this limitation does not affect the results.

However, after taking into account those limits, in conclusion, the migratory factor is not the only factor affecting the perinatal outcomes, and this study shows that the socio-professional situation, as measured by the parents’ professional income, plays a prominent role in pregnancy risk. Being in the first decile of income, compared to being in the fifth decile, leads to an increased risk of unfavourable outcomes ranging from 12% (prematurity) to 74% (very prematurity). This first decile also has a high risk of infant mortality (at the 5‰ level), which contributes to the relatively high level in Switzerland as a whole.

When economic situation is taken into account, the high risk for the children of certain categories of migrants (in particular asylum seekers or provisionally admitted migrants) is reversed, indicating that, when the financial situation is accounted for, the residence permit does not negatively influence the risk for these groups. In contrast, the children of asylum seekers and provisionally admitted women are subject to lower risks after confounding factors are accounted for.

This article provides some statistical indications on the level of risk of adverse perinatal outcomes that should be further investigated by the health system.

## Electronic supplementary material

Below is the link to the electronic supplementary material.

Supplementary material 1 (DOCX 18 kb)
