# Supplementary material for: Adverse perinatal outcomes among children in Switzerland: the impact of national origin and socio-economic group
Source: Int J Public Health. 2020 Oct 4;65(9):1613–21. doi: 10.1007/s00038-020-01492-2 (PMC7716845; doi:10.1007/s00038-020-01492-2)
Supplement: Supplementary file 1 — Supplementary material 1 (DOCX 18 kb) [file 38_2020_1492_MOESM1_ESM.docx]

**Adverse Perinatal Outcomes among Children in Switzerland. The Impact of National Origin and Socio-Economic Group**

**International Journal of Public Health**

**Supplemental Material**

*Distribution of the population according to the level of income, expressed in deciles (Switzerland 2011-2017)*

| Marital status | D1 | D2-D5 | D6-D9 | D10 | Total |
| --- | --- | --- | --- | --- | --- |
| Married (ref.) | 9.6 | 39.6 | 40.5 | 10.4 | 100.0 |
| Not married | 19.6 | 38.3 | 34.3 | 7.8 | 100.0 |
| Age of mother (ref.) |  |  |  |  |  |
| < 20 | 64.3 | 32.7 | 2.7 | 0.2 | 100.0 |
| 20-34 | 12.3 | 45.0 | 36.5 | 6.2 | 100.0 |
| 35+ | 8.6 | 24.9 | 47.1 | 19.5 | 100.0 |
| Multiple pregnancy (ref.) |  |  |  |  |  |
| No | 11.9 | 39.5 | 39.0 | 9.6 | 100.0 |
| Yes | 9.8 | 33.5 | 41.8 | 14.9 | 100.0 |
| Country of birth (ref.) |  |  |  |  |  |
| Switzerland | 6.5 | 36.5 | 48.0 | 9.0 | 100.0 |
| UE/EFTA | 10.5 | 37.0 | 36.9 | 15.6 | 100.0 |
| Other OECD | 15.8 | 19.4 | 41.0 | 23.7 | 100.0 |
| Other non-OECD | 24.2 | 48.5 | 21.0 | 6.2 | 100.0 |
| Permit of residence (ref.) |  |  |  |  |  |
| Swiss | 8.4 | 36.2 | 46.0 | 9.4 | 100.0 |
| Annual permit | 17.4 | 44.0 | 27.3 | 11.2 | 100.0 |
| Permanent permit | 10.7 | 48.5 | 30.3 | 10.5 | 100.0 |
| Asylum seeker | 88.6 | 10.9 | 0.5 | 0.0 | 100.0 |
| Other permit | 48.6 | 29.7 | 16.0 | 5.7 | 100.0 |

Sources: Swiss Population Statistics / Swiss civil register / Central Compensation Office register.

*Extract of the results of the logistic regressions on the risk of negative outcomes. Income variable, comparison of regressions conducted for 2010-2014 and 2015-2017 (Switzerland)*

|  | Infant mortality (n = 1997) | | | | Very low birth weight (n = 5797) | | | | Premature (n=46988) | | | | Extremely Premature (n = 2383) | | | |
| --- | --- | --- | --- | --- | --- | --- | --- | --- | --- | --- | --- | --- | --- | --- | --- | --- |
|  | O.R. | C.I. | | sign | O.R. | C.I. | | sign | O.R. | C.I. | | sign | O.R. | C.I. | | sign |
| 2010-2014 |  |  |  |  |  |  |  |  |  |  |  |  |  |  |  |  |
| 1st decile | 1.55 | {1.28 | -1.87} | *** | 1.38 | {1.23 | -1.55} | *** | 1.12 | {1.07 | -1.18} | *** | 1.74 | {1.45 | -2.09} | *** |
| 2nd decile | 1.13 | {0.92 | -1.38} |  | 1.15 | {1.02 | -1.30} | * | 1.04 | {0.99 | -1.09} |  | 1.37 | {1.13 | -1.66} | ** |
| 3rd decile | 1.15 | {0.94 | -1.41} |  | 1.14 | {1.01 | -1.28} | * | 1.01 | {0.97 | -1.06} |  | 1.33 | {1.09 | -1.61} | ** |
| 4th decile | 1.16 | {0.95 | -1.41} |  | 1.15 | {1.02 | -1.30} | * | 1.02 | {0.98 | -1.07} |  | 1.39 | {1.14 | -1.68} | *** |
| 5th decile (ref.) | 1.00 |  |  |  | 1.00 |  |  |  | 1.00 |  |  |  | 1.00 |  |  |  |
| 6th decile | 0.81 | {0.65 | -1.01} |  | 0.96 | {0.84 | -1.09} |  | 0.98 | {0.94 | -1.03} |  | 1.12 | {0.91 | -1.37} |  |
| 7th decile | 0.87 | {0.70 | -1.08} |  | 0.93 | {0.82 | -1.06} |  | 0.98 | {0.94 | -1.03} |  | 1.00 | {0.81 | -1.23} |  |
| 8th decile | 0.94 | {0.76 | -1.16} |  | 0.95 | {0.84 | -1.07} |  | 0.98 | {0.93 | -1.02} |  | 1.17 | {0.96 | -1.44} |  |
| 9th decile | 1.03 | {0.84 | -1.27} |  | 0.96 | {0.85 | -1.08} |  | 0.96 | {0.92 | -1.01} |  | 1.23 | {1.01 | -1.51} | * |
| 10th decile | 0.88 | {0.71 | -1.09} |  | 0.88 | {0.78 | -1.00} | * | 1.02 | {0.98 | -1.07} |  | 1.11 | {0.91 | -1.36} |  |
| 2015-2017 |  |  |  |  |  |  |  |  |  |  |  |  |  |  |  |  |
| 1st decile | 1.62 | {1.24 | -2.12} | *** | 1.35 | {1.14 | -1.60} | *** | 1.17 | {1.09 | -1.25} | *** | 1.63 | {1.26 | -2.12} | *** |
| 2nd decile | 0.96 | {0.71 | -1.31} |  | 1.15 | {0.96 | -1.38} |  | 1.04 | {0.97 | -1.12} |  | 1.04 | {0.78 | -1.40} |  |
| 3rd decile | 1.11 | {0.83 | -1.49} |  | 1.09 | {0.91 | -1.30} |  | 0.97 | {0.90 | -1.04} |  | 1.28 | {0.97 | -1.69} |  |
| 4th decile | 1.03 | {0.77 | -1.39} |  | 1.18 | {0.99 | -1.40} |  | 0.99 | {0.93 | -1.07} |  | 1.28 | {0.97 | -1.69} |  |
| 5th decile (ref.) | 1.00 |  |  |  | 1.00 |  |  |  | 1.00 |  |  |  | 1.00 |  |  |  |
| 6th decile | 0.74 | {0.54 | -1.02} |  | 0.90 | {0.74 | -1.08} |  | 0.98 | {0.91 | -1.05} |  | 0.99 | {0.74 | -1.33} |  |
| 7th decile | 0.69 | {0.50 | -0.96} | * | 0.93 | {0.77 | -1.11} |  | 0.97 | {0.90 | -1.03} |  | 0.84 | {0.62 | -1.14} |  |
| 8th decile | 0.87 | {0.64 | -1.18} |  | 0.92 | {0.76 | -1.10} |  | 0.95 | {0.88 | -1.02} |  | 1.01 | {0.75 | -1.35} |  |
| 9th decile | 0.79 | {0.58 | -1.08} |  | 0.89 | {0.74 | -1.07} |  | 0.95 | {0.88 | -1.01} |  | 1.04 | {0.78 | -1.38} |  |
| 10th decile | 0.73 | {0.52 | -1.01} |  | 0.84 | {0.70 | -1.01} |  | 1.04 | {0.97 | -1.11} |  | 0.87 | {0.64 | -1.19} |  |

Sources: Swiss Population Statistics / Swiss civil register / Central Compensation Office register.
